# Supplementary material for: Risk Assessment of the Possible Intermediate Host Role of Pigs for Coronaviruses with a Deep Learning Predictor
Source: Viruses. 2023 Jul 15;15(7):1556. doi: 10.3390/v15071556 (PMC10384923; doi:10.3390/v15071556)

Supplementary data

Supplementary Figure S1. Reduction, visualization and clustering of each type of SUI CoVs and other CoVs based on AAs, NTs and DNTs of ORF1ab and Spike sequences.

Visualization of AAs reduced with t-SNE (**A**) and PCA (**B**), and hierarchical clustering of AAs of ORF1ab of each CoV sample (**C**); Visualization of AAs reduced with t-SNE (**D**) and PCA (**E**), and hierarchical clustering of AAs of Spike of each CoV sample (**F**); Similar reduction, visualization and hierarchical clustering based on NTs of ORF1ab (**G**–**I**) and Spike (**J**–**L**) sequences; Similar reduction, visualization and hierarchical clustering based on DNTs of ORF1ab (**M**–**O**) and Spike (**P**–**R**) sequences.

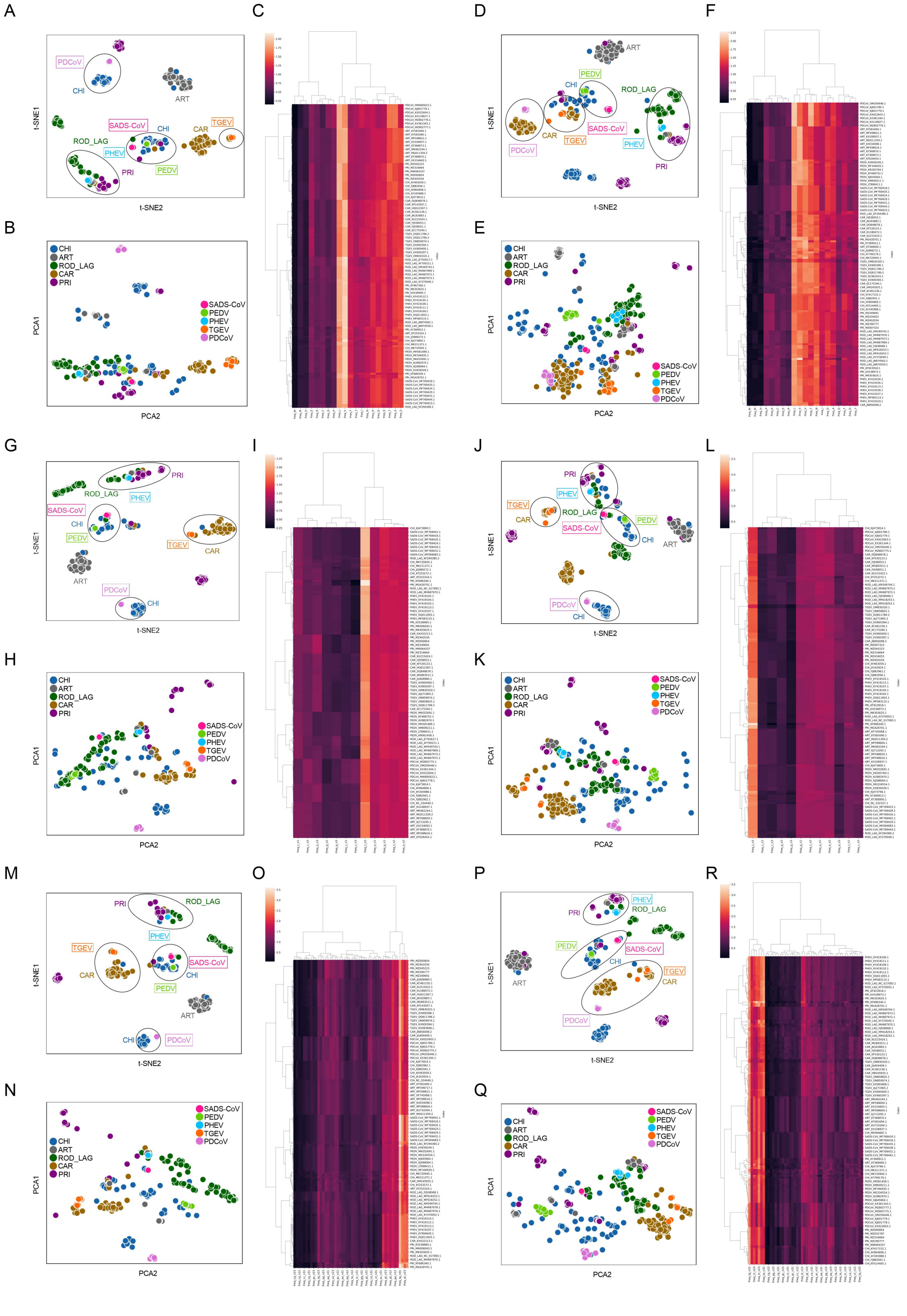
A
B
C
D
E
F
G
H
I
J
K
L
M
N
O
P
Q
R
t-SNE1
t-SNE2
PCA1
PCA2
CHI
ART
ROD_LAG
CAR
PRI
SADS-CoV
PEDV
PHEV
TGEV
PDCoV

Supplementary Figure S2. Reduction, visualization and clustering of each type of SUI CoVs and other CoVs based on codons and codonpairs of ORF1ab and Spike sequences.

Visualization of codons reduced with t-SNE (**A**) and PCA (**B**), and hierarchical clustering of codons of ORF1ab of each CoV sample (**C**); Visualization of codons reduced with t-SNE (**D**) and PCA (**E**), and hierarchical clustering of codons of Spike of each CoV sample (**F**); Similar reduction, visualization and hierarchical clustering based on codonpairs of ORF1ab (**G**–**I**) and Spike (**J**–**L**) sequences.

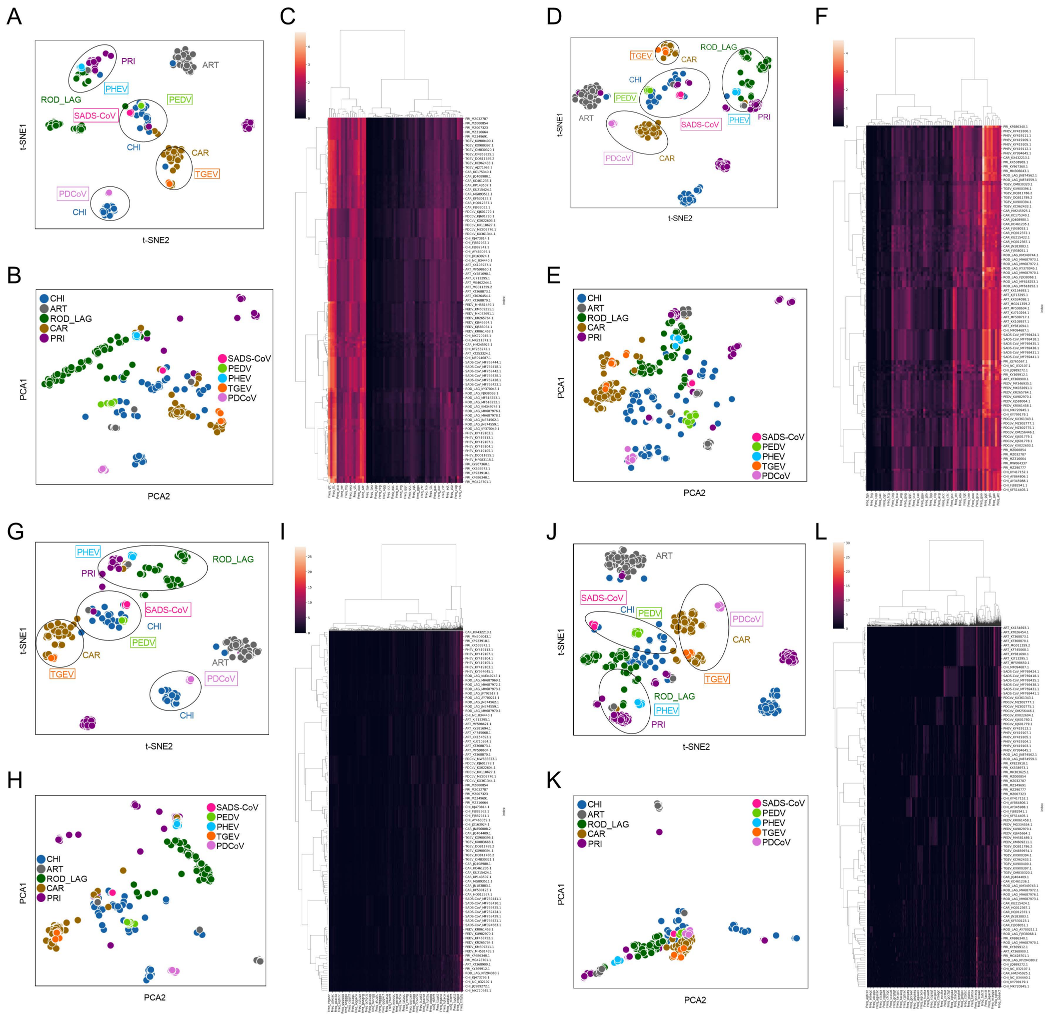

Supplementary Figure S3. Performance of the DCR-based CNN models of ORF1ab gene.

The confusion matrices (**A**), ROC with AUC (**B**), and the pair-plotting of PCA1 and PCA2 of the FC data (**C**) of the ORF1ab model with training epoch of 10, 20 (**D**–**F**), 30 (**G**–**I**), 40 (**J**–**L**), 50 (**M**–**O**). ROD means ROD_LAG.

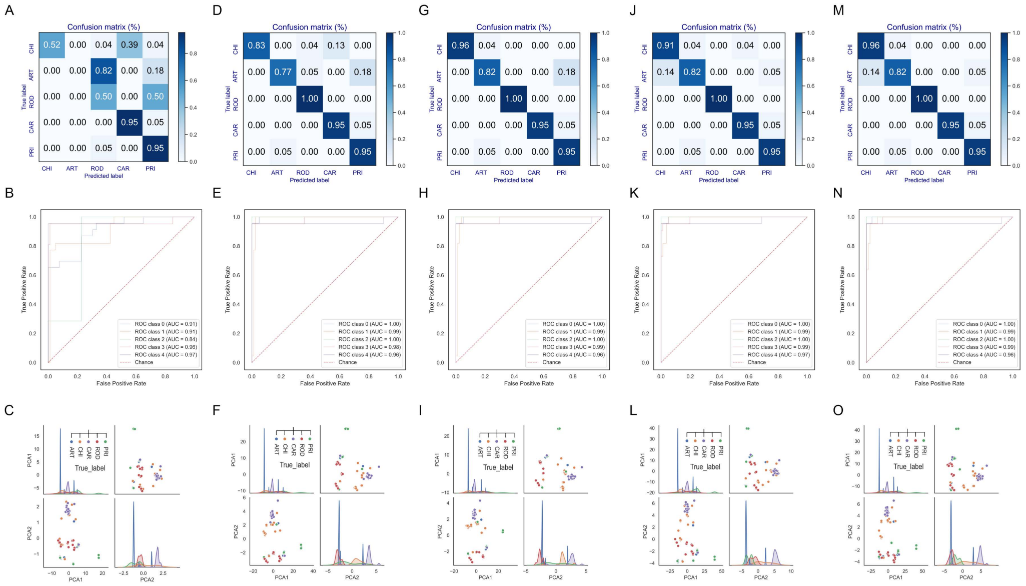

Supplementary Figure S4. Performance of the DCR-based CNN models of Spike gene.

The confusion matrices (**A**), ROC with AUC (**B**), and the pair-plotting of PCA1 and PCA2 of the FC data (**C**) of the Spike model with training epoch of 10, 20 (**D**–**F**), 30 (**G**–**I**), 40 (**J**–**L**), 50 (**M**–**O**). ROD means ROD_LAG.

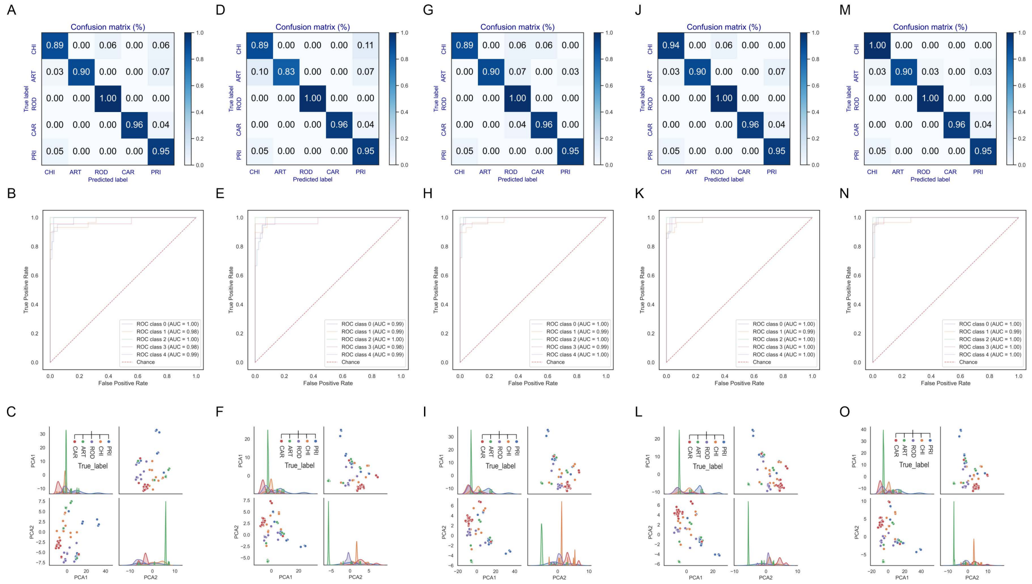

Supplementary Figure S5. Phylogenetic tree of CoVs from different adaptive hosts for ORF1ab and Spike gene.

The phylogenetic tree was constructed using iqtree with 100 bootstrap replicates for randomly sampled CoVs from CHI, ART, ROD_LAG, CAR, PRI and SUI for *ORF1ab* (**A**) and *Spike* (**B**) genes. Different colored circles in the tree represent different sample location (pink: Africa, purple: Asia, blue: Europe, green: North America, grey: Oceania). Sampling date is indicated by the progressive color bar at right.

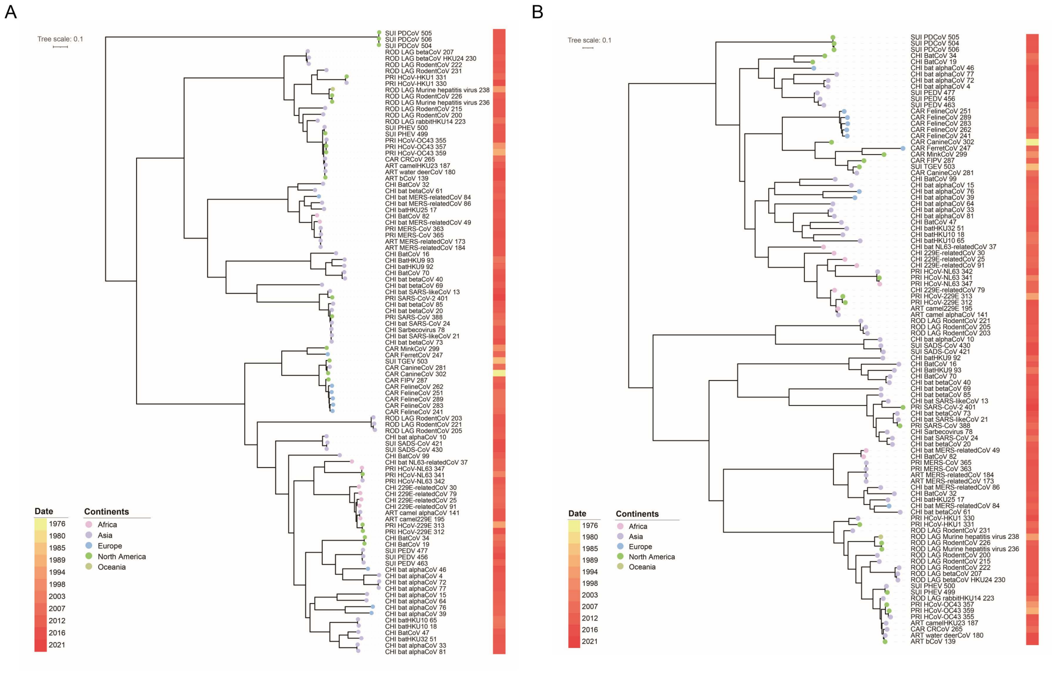

Supplementary Figure S6. Receptor binding verification of PHEV with different hosts.

Biolayer Interferometry (BLI) assay for the interaction between RBD of the PHEV Spike protein and NCAM of rabbit (**A**), rat (**B**) and human (**C**).

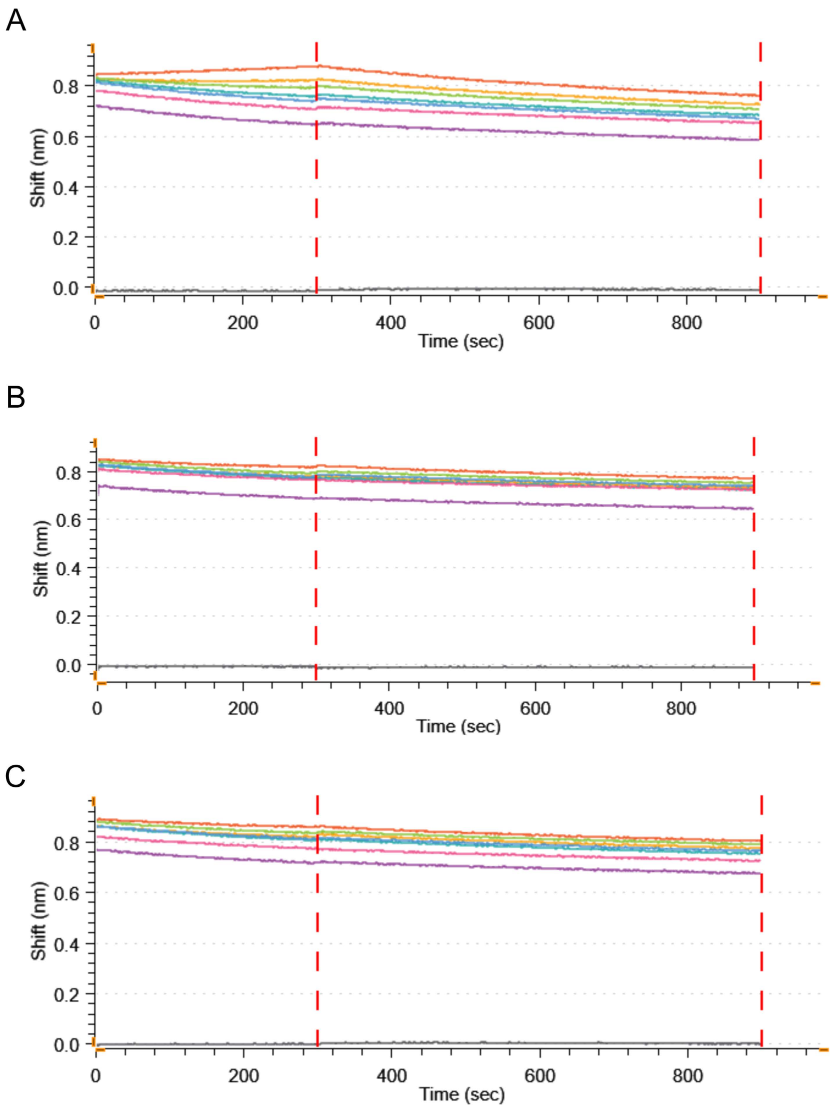

Supplement: Supplementary file 1 [file viruses-15-01556-s001.zip › viruses-2281392-supplementary.pdf]
